# Maternal and Neonatal Complications Resulting From Vacuum-Assisted and Normal Vaginal Deliveries

**DOI:** 10.7759/cureus.14962

**Published:** 2021-05-11

**Authors:** Renad A Abbas, Yasmin H Qadi, Rima Bukhari, Taghreed Shams

**Affiliations:** 1 Medicine, College of Medicine, King Saud Bin Abdulaziz University for Health Sciences, Jeddah, SAU; 2 Medicine, King Abdullah International Medical Research Center, Jeddah, SAU; 3 Clinical Research, King Abdullah International Medical Research Center, Jeddah, SAU; 4 Obstetrics and Gynecology, College of Medicine, King Saud Bin Abdulaziz University for Health Sciences, Jeddah, SAU

**Keywords:** complications, spontaneous vaginal delivery, neonatal complications, maternal complications, vacuum extraction

## Abstract

Background

Operative vaginal delivery is a procedure that is performed using forceps or vacuum to extract an infant from the birth canal. It has many indications, one of which is prolonged second stage of labor. Although rare, vacuum extraction (VE) can lead to various neonatal and maternal complications. The objective of this study was to compare the rates of different neonatal and maternal complications between vacuum-assisted deliveries and spontaneous vaginal deliveries.

Methods

This is a retrospective cohort study that was conducted in King Abdulaziz Medical City, Jeddah (KAMC-J), Saudi Arabia. The data were collected from the Labor and Delivery Unit at KAMC-J. A total of 745 samples was included (586 delivered spontaneously and 157 delivered by VE). Analysis was performed using the Statistical Package for Social Sciences (SPSS) Version 27.0.

Results

The median age was 30 years (IQR=36-34). Of all deliveries, vacuum was used in 21.1%. Perineal tear was the most frequent maternal complication (20.9%), while caput succedaneum was the commonest neonatal complication (11.8%). Post-partum hemorrhage was significantly higher among vacuum deliveries (RR=18.8; 95% CI: 5.5-64.15), as well as cephalohematoma (RR=28.9; 95% CI: 8.79-95.04) and caput succedaneum (RR=18.6; 95% CI: 10.99-31.49). The first-minute Apgar score was lower with VE (p < 0.001), and higher perineal tear degrees were reported with VE (p < 0.001).

Conclusion

The rates of maternal and neonatal complications were significantly higher among vacuum-assisted deliveries. The most serious neonatal complication was subgaleal hematoma, which is considered life-threatening. Further research is recommended to investigate subgaleal hematoma risk factors.

## Introduction

Natural birth is a spontaneous process that may or may not run smoothly. In many cases, operative delivery is required for fetal or maternal indications. Operative vaginal delivery is any method in which an instrument, whether forceps or a vacuum, is used to extract an infant from the birth canal [1]. There are many indications for operative virginal delivery, such as prolonged second stage of labor or concern about neonatal or maternal compromise [2]. Nowadays, institutions greatly rely on the use of a vacuum rather than forceps as an instrument of assisted delivery. This method is referred to as vacuum extraction (VE) where a soft or rigid suction cup adheres to the baby's head and aids in the delivery process [1]. VE is highly dependent on the traction resulting from a difference between the atmospheric and suction cup pressure as well as the pressure arising from maternal contractions and bearing down. This cumulative pressure facilitates the baby's movement through the birth canal [3]. Although rare, VE can lead to minor or major neonatal and maternal complications. In fact, 25.7% of vacuum-assisted deliveries in Belgium during the years 2001-2004 resulted in complications that required admission to the neonatal intensive care unit (NICU). Therefore, it is detrimental to measure neonatal health by the Apgar score and assess it through examination [4].

The mechanical process of VE may have an impact on neonatal outcomes. As a result, higher incidence rates of cerebrovascular disorders, spinal injuries, respiratory problems, and skull and scalp damage have been associated with several neonatal and maternal factors. Although studies regarding the topic at hand are limited, it has been proven that there is a strong correlation between vacuum-assisted deliveries and neonatal complications [4]. This, in turn, negatively affects the overall health of the infant.

The risk of complications increases due to certain factors, such as maternal height and neonatal birth weight. In 2017, an observational study was carried out in six different Swedish delivery clinics and the occurrence of neonatal complications was studied in 596 VEs. The incidence of complications was as follows: cephalohematoma (5.2%), subgaleal hematoma (1.5%), brachial plexus injury (1.3%), and severe neonatal complications (4%), which consist of intracranial hemorrhage, asphyxia, low Apgar score, convulsions, and encephalopathy. Overall 10% of infants were diagnosed to have at least one of the aforementioned neonatal complications and injuries [5]. According to a Swedish population-based cohort study published in 2014, 58% of neonatal intracranial hemorrhage are traumatic in nature due to VE and only 31.5% are spontaneous or non-traumatic in spontaneous vaginal delivery and caesarean section (CS) [6]. Additionally, in another recent study published in the United States in 2017, there was a 5% rate of neonatal complication in 555 cases of vacuum-assisted delivery, one of which had a low 5-minute Apgar score of less than 7 [7].

Since there is a gap of knowledge in studies concerning vacuum-assisted vaginal delivery, this research will contribute by enhancing the local research on this topic. Most of the available international studies target neonatal and maternal complications separately or report vacuum delivery complications with no comparison. Therefore, the objective of this study was to compare the rates of different neonatal and maternal complications between vacuum-assisted deliveries and spontaneous vaginal deliveries in King Abdulaziz Medical City in Jeddah (KAMC-J), Saudi Arabia.

## Materials and methods

Methods

This is a retrospective cohort study that aims to investigate the rates of different neonatal and maternal complications in vacuum-assisted deliveries in comparison to spontaneous vaginal deliveries. A total of 745 samples were included (586 delivered spontaneously and 157 delivered by VE). Data were collected from the Labor and delivery Unit at KAMC-J. The collected data included all vacuum-assisted vaginal deliveries and spontaneous vaginal deliveries. On the other hand, the exclusion criteria are all medical records that have missing data, attempted VE that ended up with performing CS, and the combined use of assisted vaginal delivery that utilizes forceps.

Data collection

The delivery room records were reviewed to identify the medical record numbers of women who delivered by VE and spontaneous vaginal delivery. Cases between June 2016 and December 2018 were included in the study. Data regarding maternal and neonatal variables were collected based on a data abstraction sheet using the health information system (BESTCare) at the National Guard Hospital in KAMC-J. The sheets included both maternal and neonatal variables. Maternal variables were age, weight, height, BMI, parity, gravidity, gestational age, nature of labor, mode of delivery, and the presence of perineal tear, postpartum hemorrhage, or any other complications. Neonatal variables were gender, weight, Apgar scores, site of admission, and the presence of cephalohematoma, scalp laceration, subgaleal hematoma, brachial plexus injury, or any other complications.

Statistical analysis

The Statistical Package for the Social Sciences (SPSS) Version 27.0 (IBM Corp., Armonk, NY, USA) was used to analyze the data. Missing values were replaced in variables missing less than 10%. Median for nearby points was the method used to replace the missing numerical variables. The median and interquartile range (IQR) were used to summarize the numerical variables, while the proportion was used for categorical data. Non-parametrical tests, Mann-Whitney and chi-square tests, were used to find significance. Binary logistic regression was used to investigate the effect of numerical data on binary outcome variables. Linear regression was skipped due to the large skewness of the outcome variable. P-values of less than 0.05 were considered to be significant, and 95% confidence level was used for inferential statistics.

## Results

A total of 787 patients were enrolled and analyzed. The median (M) age was 30 years (IQR=26-34). The medians were calculated for body mass index (BMI) (M=28.32 kg/m^2^; IQR=24.75-31.88), gravidity (M=4; IQR=2-5), parity (M=2; IQR=1-4), and gestational age at delivery (M=39 weeks; IQR=38-40). Moreover, the weight of the newborn had a median of 3.04 months (IQR=2.79-3.35). As for the newborn sex, 54.7% were males, 45.2% were females, and one newborn 0.1% was ambiguous.

The majority (79.6%) had spontaneous delivery, while the induced labors represented 20.4%. Regarding the mode of delivery, the vacuum method was utilized in 21.1% of the deliveries, while the rest were normal spontaneous vaginal deliveries. The majority 89.9% of newborns were admitted for nursery care, 7.6% needed NICU, and 2.5% were taken to the hospital morgue. As for the complications, maternal complications included post-partum hemorrhage and perineal tear, while the neonatal included a variety of complications. As for neonatal complications, no cases of brachial plexus injury were documented. Of the newborns, 10.1% had nuchal cord, 4.1% had respiratory distress, 1.3% had shoulder dystocia, and 1.1% had other complications. However, other complications are shown in Table 1.

**Table 1:** General maternal and neonatal complications all deliveries included in the study

Complication	Proportion
Maternal complications	
First-degree perineal tear	6.8%
Second-degree perineal tear	9.3%
Third-degree perineal tear	2%
Fourth-degree perineal tear	0.8%
Unspecified degree perineal tear	2%
Post-partum hemorrhage	2.4%
Neonatal complications	
Cephalohematoma	3.4%
Scalp laceration	0.8%
Subgaleal hematoma	0.4%
Brachial plexus injury	0%
Caput succedaneum	11.8%
Others	16.7%

The statistical differences between normal spontaneous vaginal delivery (NSVD) and vacuum-assisted deliveries in continuous variables were tested. The mean rank for perineal tear degree was significantly higher among those who underwent vacuum-assisted delivery (560.8), while the mean rank was 331.38 for NSVD (p ≤ 0.001). The first-minute Apgar score was significantly lower among vacuum-assisted than NSVD group (315 and 399.31, respectively) (p ≤ 0.001). Nevertheless, five-minute Apgar score and duration of stay in the hospital were not significantly different between the two groups. Categorical variables were tested to find differences between the two modes of delivery. While site of admission and subgaleal hematoma failed to show any significance, other significant associations with the correlated relative risk and 95% confidence intervals are shown in Table 2. Binary logistic regression was done independently to find the effect of continuous variables on maternal and neonatal complications. The majority did not show any significant effect. The complications that showed a significant regression on the continuous variables are shown in Table 3.

**Table 2:** Crosstabulation showing significant the effect of the delivery method on complications *Significance was measured using the chi-square test NSVD, normal spontaneous vaginal delivery; RR, relative risk

	Vacuum-assisted delivery	NSVD	RR (95% CI)	p-Value*
Post-partum hemorrhage	15 (9.4%)	3 (0.5%)	18.8 (5.51-64.15)	<0.001
Cephalohematoma	23 (14.6%)	3 (0.5%)	28.9 (8.79-95.04)	
Scalp laceration	6 (3.8%)	0 (0%)	-	<0.001
Subgaleal hematoma	2 (1.3%)	1 (0.2%)	7.54 (0.69-82.62)	0.051
Caput succedaneum	74 (47.1%)	15 (2.5%)	18.6 (10.99-31.49)	<0.001

**Table 3:** The significant regression of maternal and neonatal complications on the numerical variables *Significance was measured independently using binary logistic regression

Variable	Odds (95% CI)	p-Value*
Post-partum hemorrhage		
Gravidity	0.59 (0.43-0.83)	0.002
Parity	0.48 (0.31-0.74)	<0.001
Cephalohematoma		
Gravidity	0.67 (0.52-0.86)	0.002
Parity	0.56 (0.41-0.77)	<0.001
Scalp laceration		
Gravidity	0.32 (0.12-0.87)	0.025
Parity	0.14 (0.02-0.86)	0.034
Caput succedaneum		
Age	0.91 (0.88-0.95)	<0.001
Gravidity	0.52 (0.43-0.61)	<0.001
Parity	0.42 (0.34-0.52)	<0.001
Abortion	0.68 (0.48-0.98)	0.036

## Discussion

The vacuum delivery prevalence in this study was found to be 21.1%. While it is not favorable to have one vacuum-assisted delivery out of five deliveries, it is considered lower compared to a study that was carried out in France, which reported a prevalence of 29.7% vacuum-assisted deliveries [8]. The high prevalence of complications could be related to the fact that VE is used for various indications; nonetheless, it is considered the first operative attempt when dealing with second-stage events [9]. However, there are various options for an operative vaginal delivery without having to perform a CS. Vacuum and forceps are considered the most common methods used, with a tendency towards using vacuum more frequently than forceps [10]. This preference can be explained by the fact that in comparison to forceps, vacuum is associated with lower risks of maternal and neonatal complications [11,12]. Multiple other studies showed that forceps are associated with a higher rate of maternal complications, while vacuum-assisted delivery is associated with higher fetal morbidities [13]. This study revealed that vacuum was significantly associated with maternal and neonatal complications. The most common neonatal complication among our sample was chignon. Chignon, “also known as caput succedaneum”, is a temporary anatomical defect that would typically disappear in the first 24 hours after birth and has no clinical long-term effects [14]. Another frequently reported complication is scalp laceration. While less than 1% suffered from scalp laceration in this study, the literature reported a higher prevalence that can reach 82% [14]. The absence of a clear definition for scalp laceration is thought to be the reason for the great gap between the reported percentages [14]. However, despite the high rate of a chignon, this study agrees with the literature that most complications are comprised of cephalohematomas followed by subgaleal hematoma then brachial plexus injuries [5]. The most commonly reported maternal complication is perineal tear, which was also high among our population (reaching 20.9%), with second-degree perineal tear as the most frequent subtype (9.3%). Admission to the NICU was considered a complication in the present study, where 7.6% of the neonates included in this study required admission to the NICU. In addition, hematoma was found in 3.4%. The previous complications are lower than the rates reported from a study conducted in Belgium, which reported rates of 25.6% and 10.8% for NICU admission and cephalohematoma, respectively [4]. Several factors were found to be associated with lower rates of maternal and neonatal complications. Interestingly, a number of gravidity and parity were found to be protective against postpartum hemorrhage. Furthermore, statistically, higher gravidity and parity were also protective against three neonatal complications: cephalohematoma, scalp laceration, and chignon. This, in summary, can indicate a reverse relationship between the number of previous conceptions and the rate of complications. However, inferences for some complications were not obtained due to the low number or absence of the event such as subgaleal hematoma and brachial plexus injury. Most of the neonatal complications are not serious and would resolve with no intervention needed such as cephalohematoma, lacerations, and chignon [15]. On the other hand, subgaleal hematoma is considered a serious and life-threatening neonatal complication. Although rare, mortalities approach 25% of the affected neonates [14]. The results of the present study address clearly that vacuum-assisted delivery is associated with greater risk of complications compared to NSVD. This finding was also supported by a retrospective study conducted by Baume et al., where perineal tear, cephalohematoma, and scalp lacerations were significantly higher among the vacuum-assisted group [16,17]. The same study concluded that vacuum use is considered an advantage despite the infrequent complications reported [17]. Whenever operative delivery is indicated, indeed, vacuum is considered a better option than forceps [18]. Our study has strengths and limitations. The strength of this study is represented in the comparison between vacuum-assisted delivery and NSVD, in which the latter was used as a control group. Taking advantage of this allowed performing a detailed unbiased analysis of the factors addressed in the literature. The hospital-based design is considered a limitation, in which the generalizability of the results is not possible. Other limitations include missing data and zero cases of some complications. Thus, no inferential statistics could have been performed.

## Conclusions

Vacuum-assisted delivery is associated with more maternal and neonatal complications than normal vaginal delivery. Perineal tear was the most frequent maternal complication, whereas caput succedaneum was the commonest neonatal complication. Maternal gravidity and parity were found to be protective against postpartum hemorrhage, cephalohematoma, subgaleal hematoma, and chignon. Most neonatal complications are temporary with no long-term effects, except for subgaleal hematoma. Therefore, we recommend performing further case-control studies to address the associated risk factors for subgaleal hematoma.
